# Clinical relevance of reduced decorrelation signals in the diabetic inner choroid on optical coherence tomography angiography

**DOI:** 10.1038/s41598-017-05663-9

**Published:** 2017-07-12

**Authors:** Yoko Dodo, Kiyoshi Suzuma, Kenji Ishihara, Shin Yoshitake, Masahiro Fujimoto, Tatsuya Yoshitake, Yuko Miwa, Tomoaki Murakami

**Affiliations:** Department of Ophthalmology and Visual Sciences, Kyoto University Graduate School of Medicine, Kyoto, Japan

## Abstract

Diabetes induces lesions of the retinal and choroidal capillaries, which promote the pathogenesis of diabetic retinopathy (DR). The decorrelation signals in optical coherence tomography angiography (OCTA) represent the blood flow and vascular structure, and three-dimensional OCTA images enable individual capillary layers to be evaluated separately. The current study documented that en-face OCTA images revealed spots of flow void in the choriocapillaris layer in eyes with DR. Quantitative investigation demonstrated that non-flow areas within the central subfield (CSF) increased in eyes with more severe DR grades. The non-flow areas in the choriocapillaris layer were also associated with poorer visual acuity (VA) in all 108 eyes. A modest correlation was noted between the areas of flow void and poorer VA in 69 eyes without DME, whereas the non-flow areas were not related to VA or to CSF thickness measured by OCT in 39 eyes with DME. In 12 eyes with ischemic maculopathy, the choriocapillaris layer beneath the disrupted ellipsoid zone of the photoreceptor (EZ) had greater areas of flow void than did the area beneath an intact EZ. These data suggested that disrupted choroidal circulation has clinical relevance and contributes to the pathogenesis of DR.

## Introduction

Diabetic retinopathy (DR), which is a diabetic microangiopathy, often leads to severe visual impairment^1^. Therapeutic strategies, including anti-vascular endothelial growth factor (VEGF) therapy, photocoagulation, and vitrectomy, have been widely used to treat vision-threatening DR, e.g., proliferative diabetic retinopathy (PDR) and diabetic macular edema (DME). However, patients with diabetes often have poor visual prognosis^2–4^. Further investigations should elucidate the pathogenesis in the chorioretinal vessels and its effect on neuroglial dysfunction^5, 6^.

In the physiologic state, the inner and outer retinal layers are nourished via the retinal and choroidal vessels, respectively, and diabetes impairs the function and structure of these vessels. The choroidal vessels are composed of hierarchical structures that are represented by their layers. From the short posterior ciliary arteries, larger and smaller vessels in Haller’s layer and Sattler’s layer bifurcate into fine meshwork-like capillaries with small intercapillary spaces in the choriocapillaris layer^7–9^. The choroidal system nourishes the outer retinal layers in concert with the retinal pigment epithelium (RPE) and Bruch’s membrane^10^. A few histologic publications have documented the morphologic changes in the choroidal vasculature and periodic acid-Schiff positive deposits in the stroma and have proposed “diabetic choroidopathy” as another diabetic microangiopathy^11, 12^. In addition to the arteriosclerotic changes or vascular luminal narrowing in the larger choroidal vessels, capillary dropout in the choriocapillaris layer suggests ischemic changes in the outer retinal layers^13^. However, the *in vivo* relationship between the choriocapillaris and the outer retinal layers has not been investigated in diabetic eyes^14^.

Recent advances in fundus imaging have promoted the understanding of the pathogenesis in DR. Enhanced-depth imaging of spectral-domain optical coherence tomography (OCT) and swept-source OCT have particularly enabled both the qualitative and the quantitative *in vivo* evaluation of choroidal structure^15, 16^. Whether the quantified choroidal thickness is associated with DR severity or DME remains controvesial^17–24^. Recent publications have documented several morphologic changes in the choroidal vasculature in Sattler’s layer or Haller’s layer in diabetic eyes^25, 26^. OCT angiography (OCTA), in which the decorrelation signals derive mainly from the movements of erythrocytes, delineates the three-dimensional vascular structure^27–30^. In healthy eyes, the decorrelation signals are depicted in the inner choroidal layers, whereas the signals decrease or disappear in the lesions of several chorioretinal diseases^31, 32^. However, it is largely unknown how OCTA delineates the flow void in the choriocapillaris layer in eyes with DR.

In the current study, we evaluated the decorrelation signals in the choriocapillaris layer on OCTA images and their association with DR severity and pathogenesis in retinal lesions in DR.

## Results

### Characteristics of decorrelation signals in the choroidal capillary slab in diabetic eyes

**Figure 1:**
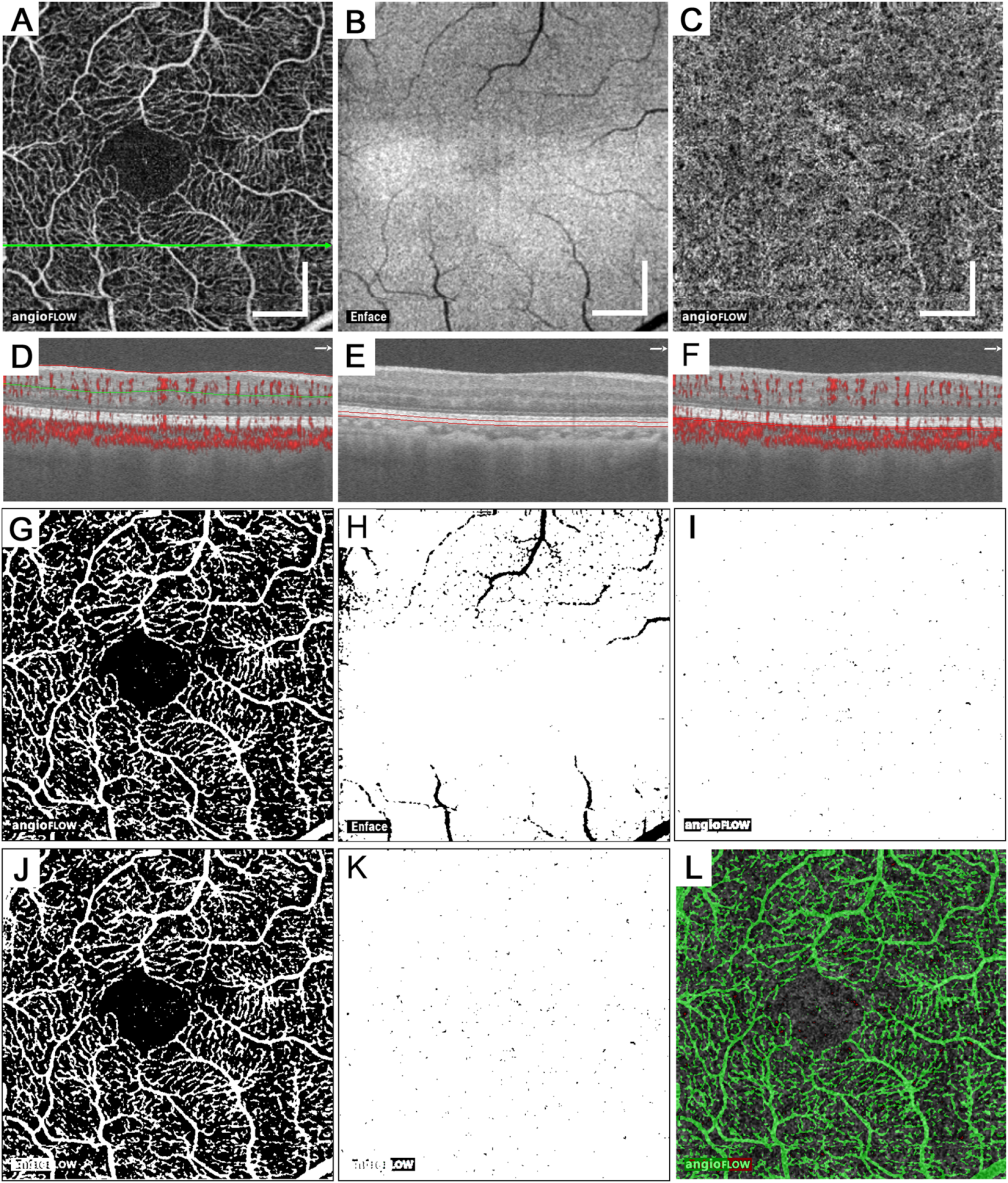
Tiny dots of flow void in the choriocapillary layer in a 60-year-old patient with no apparent retinopathy. The en-face OCTA images in the superficial retinal layer (**A**) and the choriocapillaris layer (10 μm thick) (**C**) and structural OCT image at the RPE level (**B**). The corresponding B-scan images along the green arrow in panel A with (**D**,**F**) or without (**E**) the decorrelation signals (red). Red or green lines revealed the segmentation of individual layers. (**G**,**H**) The binary images of panels A and B using the global threshold in ImageJ. (**I**) The binary image of panel C using the mean decorrelation signal in the outer retina as the cutoff value. (**J**) The merged image of panel H with the inverted signal and panel G means the projection artifacts and/or shadow artifacts. (**K**) The areas of flow void in the choriocapillaris layer (black) after removing the artifacts. (**I**) The merged image of panels C (grayscale), (**J**) (green), and (**K**) with inverted signals (red). Scale bar = 500 μm.

**Figure 2:**
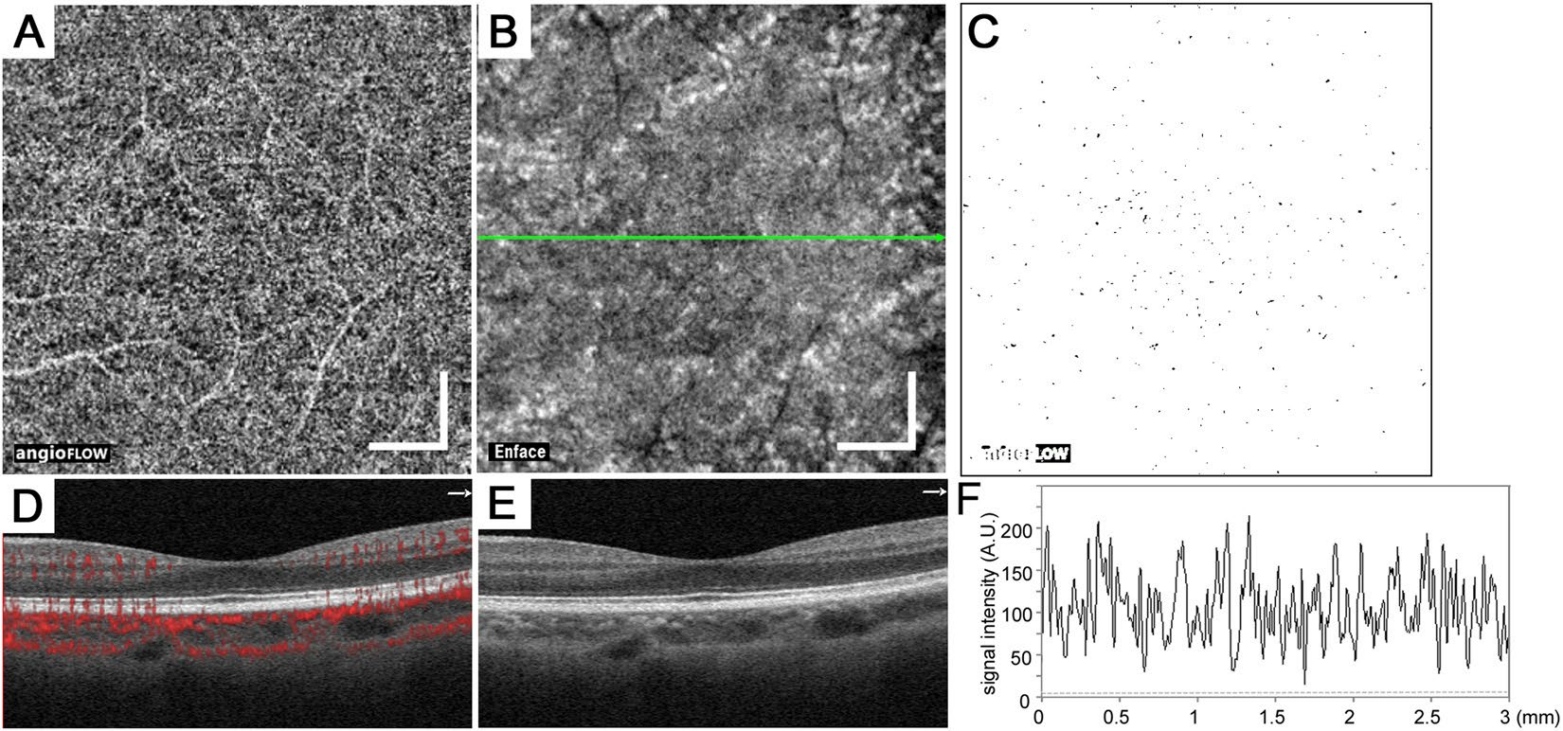
The mosaic with higher or lower intensity decorrelation signals in the choriocapillaris layer of a healthy eye. The 10-μm-thick en-face OCTA (**A**) and OCT (**B**) images in the choriocapillaris layer. (**C**) A binary image reveals the areas of flow void (black) after the removal of the artifacts. B-scan image with (**D**) and without (**E**) decorrelation signals along the green arrow in (**B**). (**F**) The intensity of the decorrelation signal along the green line in B. Scale bar = 500 μm.

**Figure 3:**
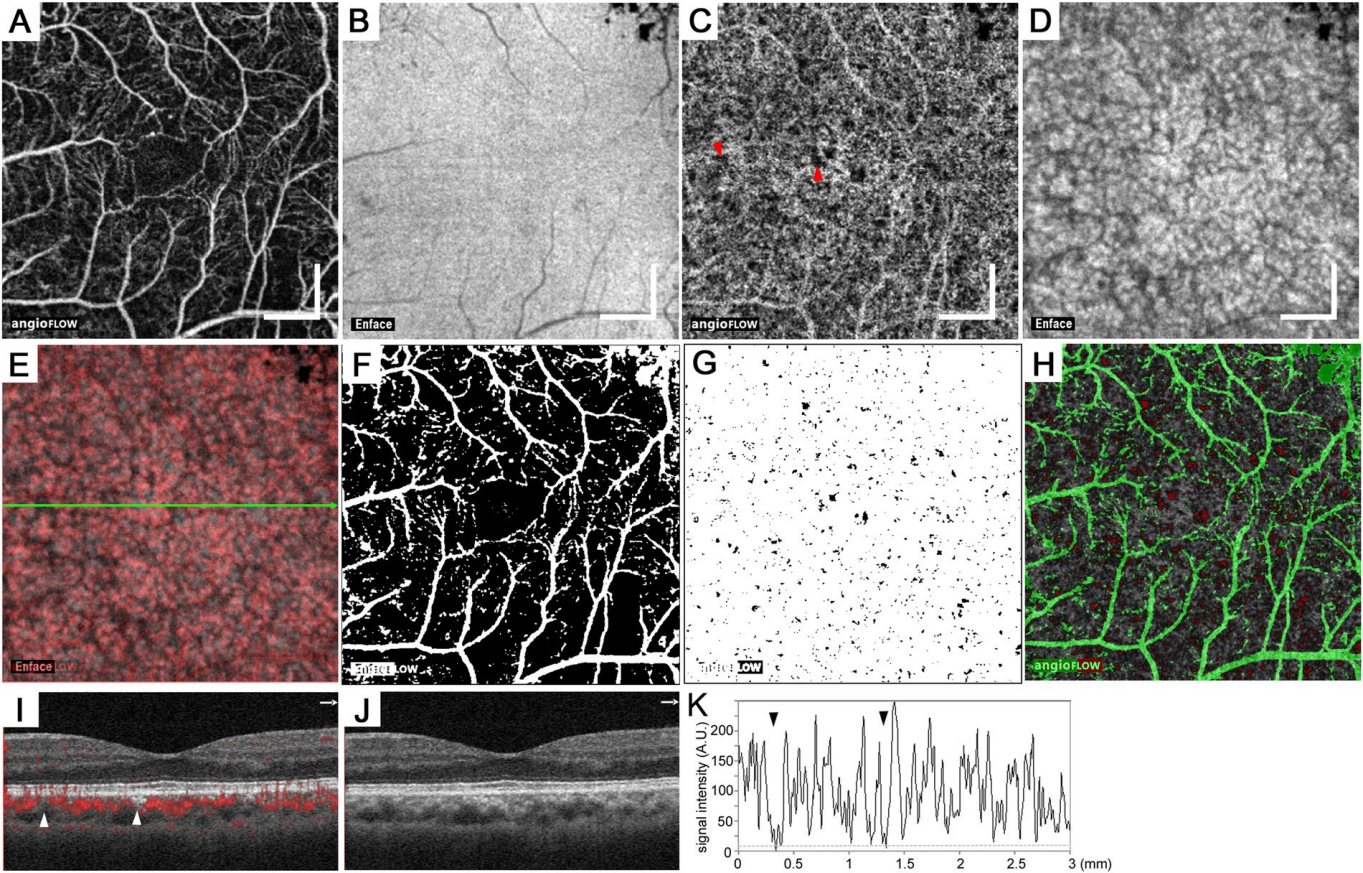
Spots of flow void in the choriocapillaris layer of a 53-year-old patient with severe NPDR. The en-face OCTA images in the superficial retinal layer (**A**) and the 10-μm-thick choriocapillaris layer (**C**) and the structural OCT images in the RPE (**B**) and the choriocapillaris layer (**D**). (**E**) The merged image of panels C (red) and D (grayscale). (**F**) The en-face binary image of projection and/or shadow artifacts created from panels A and B. (**G**) The binary images of the non-flow areas (black) in the choriocapillaris layer after artifact removal. (**H**) The merged image of panel C (grayscale), (**F**) (green), and (**G**) with inverted signals (red). (**I**,**J**) B-scan images with and without decorrelation signals (red) along the green arrow in panel E. (**K**) The intensity of the decorrelation signals along the green arrow in panel E. The arrowheads in panels C,I, and K indicate spots of flow void. Scale bar = 500 μm.

**Figure 4:**
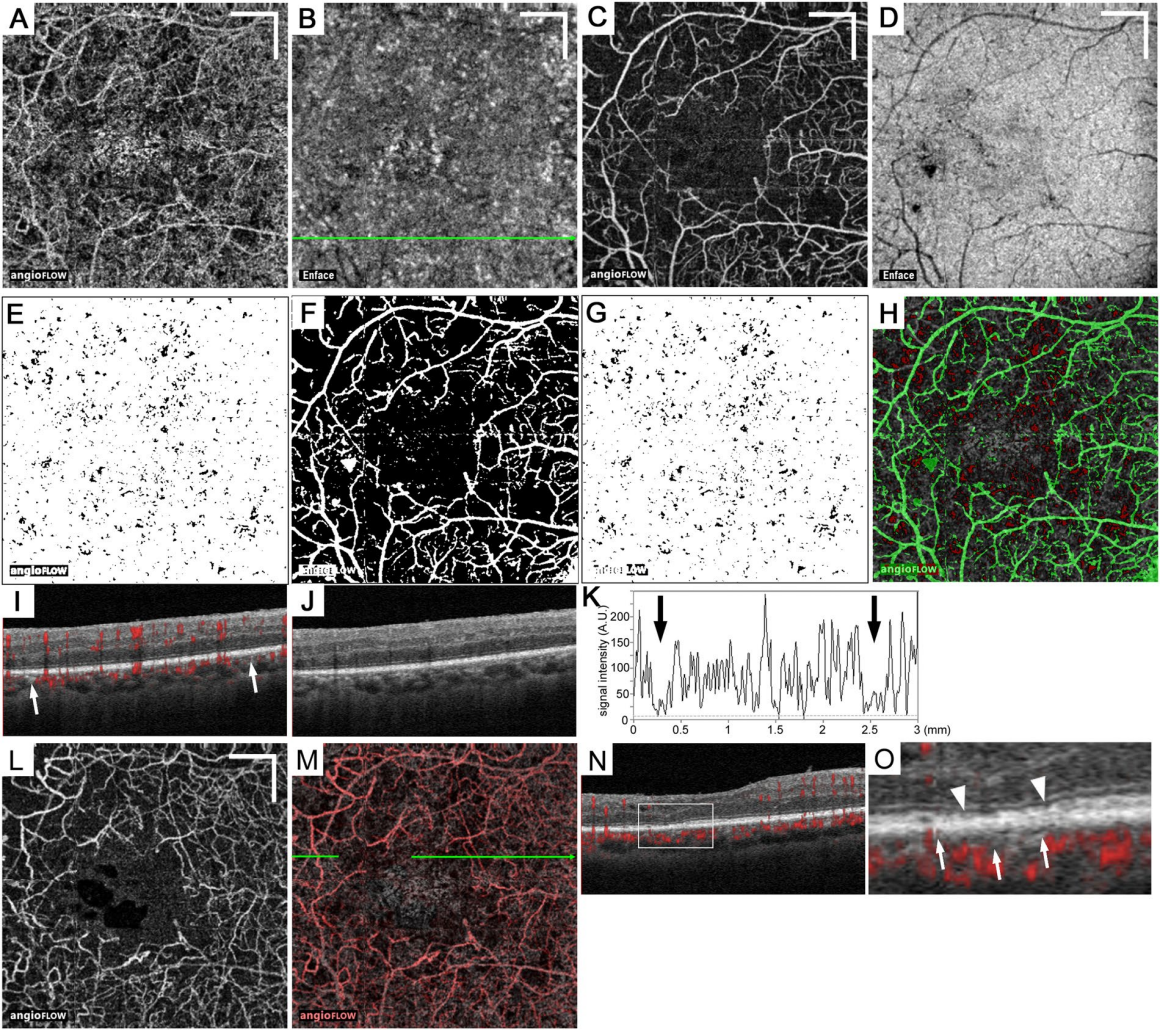
Larger lesions with reduced decorrelation signals in the choriocapillaris slab in a 68-year-old patient with PDR. (**A**,**B**) The 10-μm-thick en-face OCTA and structural OCT images in the choriocapillaris layer. The en-face OCTA image in the superficial retinal layer (**C**) and the structural OCT image at the RPE level (**D**). (**E**) The binary image of panel A using the mean decorrelation signal in the outer retina as the cutoff value. (**F**) The binary image of the projection artifacts and/or shadow artifacts created from panels C and D. (**G**) The binary image of the areas of flow void after the removal of the areas with the artifacts. (**H**) The merged image of panel A (grayscale), (**G**) with inverted signals (red), and the binary image of artifacts (**F**; green). (**I**,**J**) B-scan images with and without decorrelation signals (red) along the green arrow in (**B**). (**K**) The intensity of the decorrelation signals along the green arrow in (**B**). (**L**) The en-face OCTA image in the deep retinal capillary layer. (**M**) The merged image of panels A (grayscale) and L (red). (**N**,**O**) The sectional image along the green arrow in panel M and its magnified image demonstrate that the EZ line is not intact in the areas of flow voids in both the retinal and the choroidal capillaries (between the arrowheads). (**O**) The white or black arrows in (**I**,**K**, and **O**) indicate the areas of flow void in the choriocapillaris layer. Scale bar = 500 μm.

**Figure 5:**
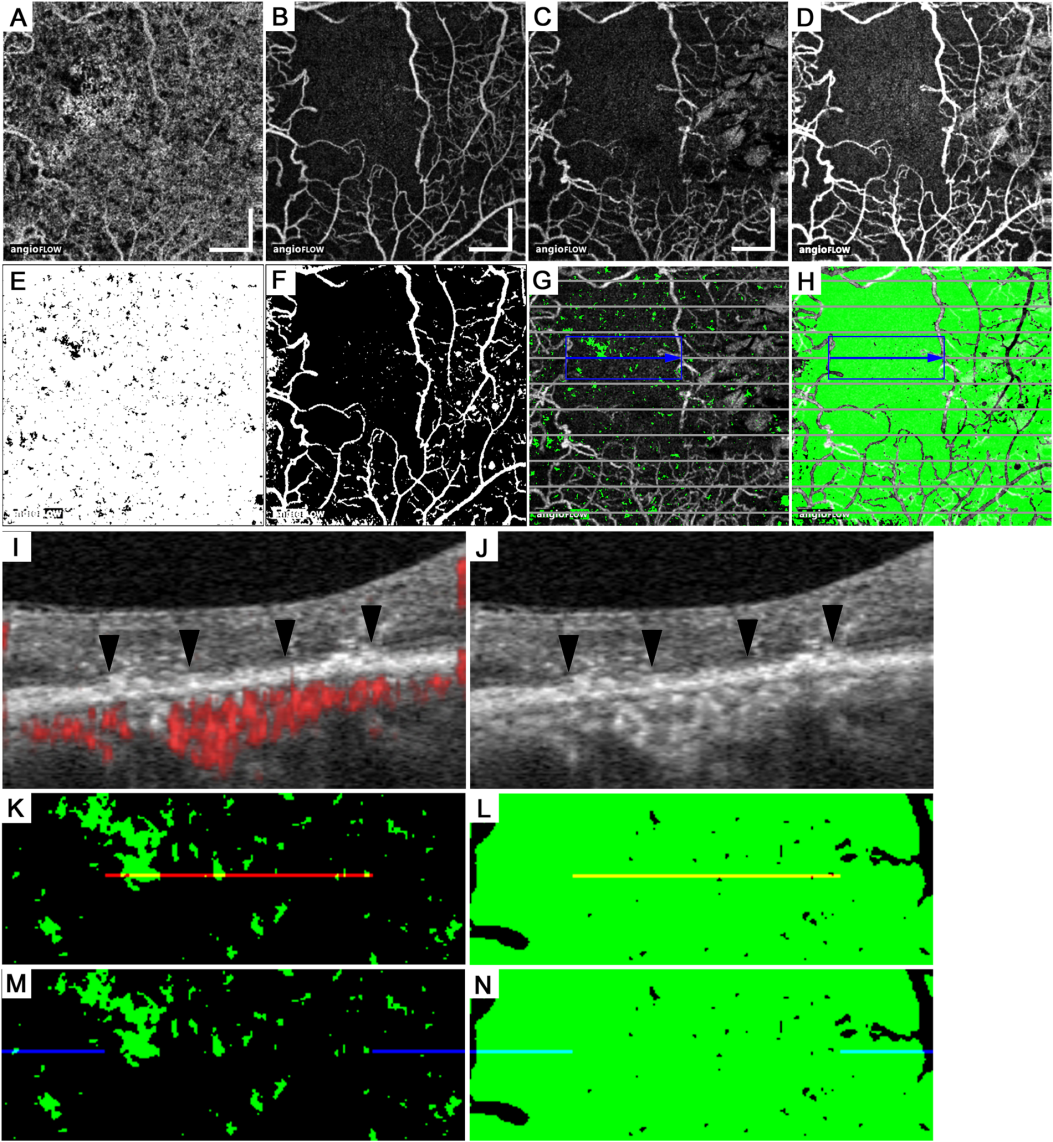
Association between photoreceptor damage and flow void in the choriocapillaris layer in a 46-year-old patient with PDR. En-face OCTA images in the 10-μm-thick choriocapillaris layer (**A**) and the superficial (**B**) and deep (**C**) retinal capillary layers. (**D**) The merged image of the superficial and deep layers reveals ischemic maculopathy. The binary images of the flow void (**E**) or the artifacts (**F**). (**G**,**H**) The merged images of the deep capillary layer (panel C) and panel E or F with inverted signals (green). Both EZ status and decorrelation signals in the choriocapillaris layer were evaluated along the 10 gray lines. (**I**,**J**) The B-scan images with and without decorrelation signals (red) along the blue arrow in panels G and H demonstrate that the EZ line is not intact in the central region (arrowheads). The magnified images of areas of flow void (green; **K**,**M**) and those without the artifacts (green; **L**,**N**) within the blue rectangle in panels G and H. (**K**,**L**) The red lines correspond to the ‘not intact’ EZ line in panel I and partly colocalize with the areas of flow void. (**M**,**N**) The blue lines correspond to the ‘intact’ EZ line in panel I and rarely colocalized with the non-flow areas. Scale bar = 500 μm.

After excluding 93 eyes that met the exclusion criteria, 108 eyes of 66 consecutive patients with diabetes mellitus were evaluated. The patients’ characteristics are presented in Table 1. Qualitative investigation indicated that the OCTA images revealed that the mosaic of the higher decorrelation signal was accompanied by tiny dots with lower signal intensity in the en-face choroidal capillary images in eyes with no apparent retinopathy (Figs 1 and 2). By contrast, spots of flow void were often observed in eyes with nonproliferative DR (NPDR) or PDR (Fig. 3). Eyes with moderate NPDR or with more severe grades often exhibited larger lesions of flow void. These features were accompanied by a vessel-like flow signal, which might correspond to either choroidal vessels or projection artifacts from retinal vessels (Figs 4 and 5).

**Table 1:** Patient characteristics.

Characteristic	No apparent retinopathy	Mild NPDR	Moderate NPDR	Severe NPDR	PDR
Eyes/patients	11/6	8/5	47/31	15/11	27/17
Age (years)	64.8 ± 11.7	71.5 ± 19.6	67.1 ± 10.9	60.7 ± 9.8	52.9 ± 12.4
Men/women	5/1	3/2	24/7	9/2	12/5
Diabetes duration (years)	13.7 ± 2.3	13.0 ± 2.9	9.8 ± 6.9	14.2 ± 7.4	9.8 ± 8.1
HbA1c (%)	7.33 ± 1.91	6.75 ± 0.35	7.61 ± 1.77	7.54 ± 0.78	7.57 ± 1.38
Systemic hypertension (present/absent)	2/4	5/0	18/13	7/4	11/6
Dyslipidemia (present/absent)	1/5	2/3	10/21	3/8	4/13
LogMAR VA	−0.033 ± 0.175	0.095 ± 0.212	0.038 ± 0.159	0.068 ± 0.184	0.104 ± 0.258
Center-involved DME	—	4 eyes	14 eyes	10 eyes	11 eyes
Ischemic maculopathy	—	0 eye	0 eye	6 eyes	6 eyes
CSF thickness (µm)	244 ± 36	290 ± 58	284 ± 61	346 ± 69	325 ± 120
SSI	70.8 ± 4.9	68.5 ± 5.8	67.3 ± 5.4	66.2 ± 4.7	70.0 ± 6.6

Further quantification demonstrated that the areas of flow void did not differ among the central subfield (CSF) and the superior, nasal, temporal, and inferior subfields in the parafovea of the modified Early Treatment Diabetic Retinopathy Study (ETDRS) grid (3.25 ± 1.85%, 2.93 ± 1.69%, 2.91 ± 1.53%, 3.00 ± 1.76%, and 3.04 ± 1.55%, respectively; *p* = 1.000 for all comparisons) after the projection artifacts and shadow artifacts were carefully removed (Fig. 1).

### Clinical relevance of flow void in the choroidal capillary slab

**Figure 6:**
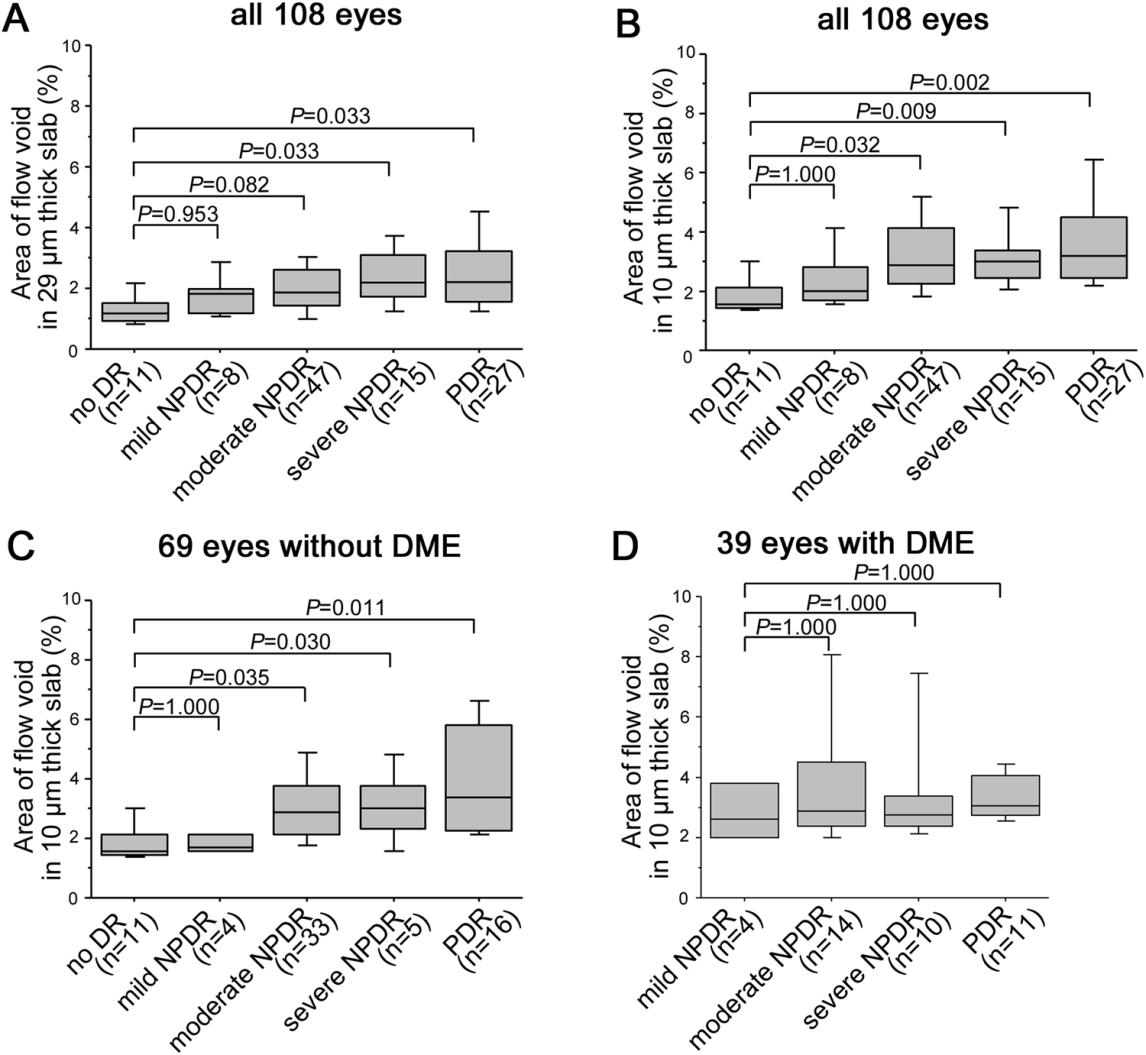
The areas of flow void in the choroid capillary OCTA slab in individual DR severity grades. The non-flow areas within the CSF in the 29-μm-thick choroid capillary slab of the default setting (**A**) or the 10-μm-thick choriocapillaris layer (**B**) increase gradually according to individual DR severity grades in all 108 eyes. Areas of flow void in the 10-μm-thick choriocapillaris layer in eyes without (**C**) and with (**D**) center-involved DME.

We evaluated how the areas of flow void in the CSF were associated with systemic and ocular parameters. We prepared the en-face OCTA images of choroidal capillary slab (~29 μm thickness) according to the default setting (Fig. 6A). Eyes with severe NPDR or PDR had greater areas of flow void than those with no apparent retinopathy (Fig. 6A). On the ~10-μm-thick choriocapillaris slab images, the areas increased gradually according to the DR severity, and eyes with moderate NPDR, severe NPDR, and PDR had significantly larger areas of flow void than those with no apparent retinopathy (*p* = 0.032, *p* = 0.009, and *p* = 0.002, respectively) (Fig. 6B). We selected the non-flow areas in the 10-μm-thick slab images for further evaluation to investigate the clinical relevance of the flow void in the choriocapillaris layer. In 69 eyes without center-involved DME, the areas of flow void tended to increase as the DR severity progressed, and eyes with severe NPDR or PDR had larger non-flow areas than those with no apparent retinopathy (Fig. 6C). By contrast, we found no associations of the non-flow areas with systemic factors, i.e., age, hemoglobin A1c, diabetes duration, systemic hypertension, or dyslipidemia (data not shown).

**Figure 7:**
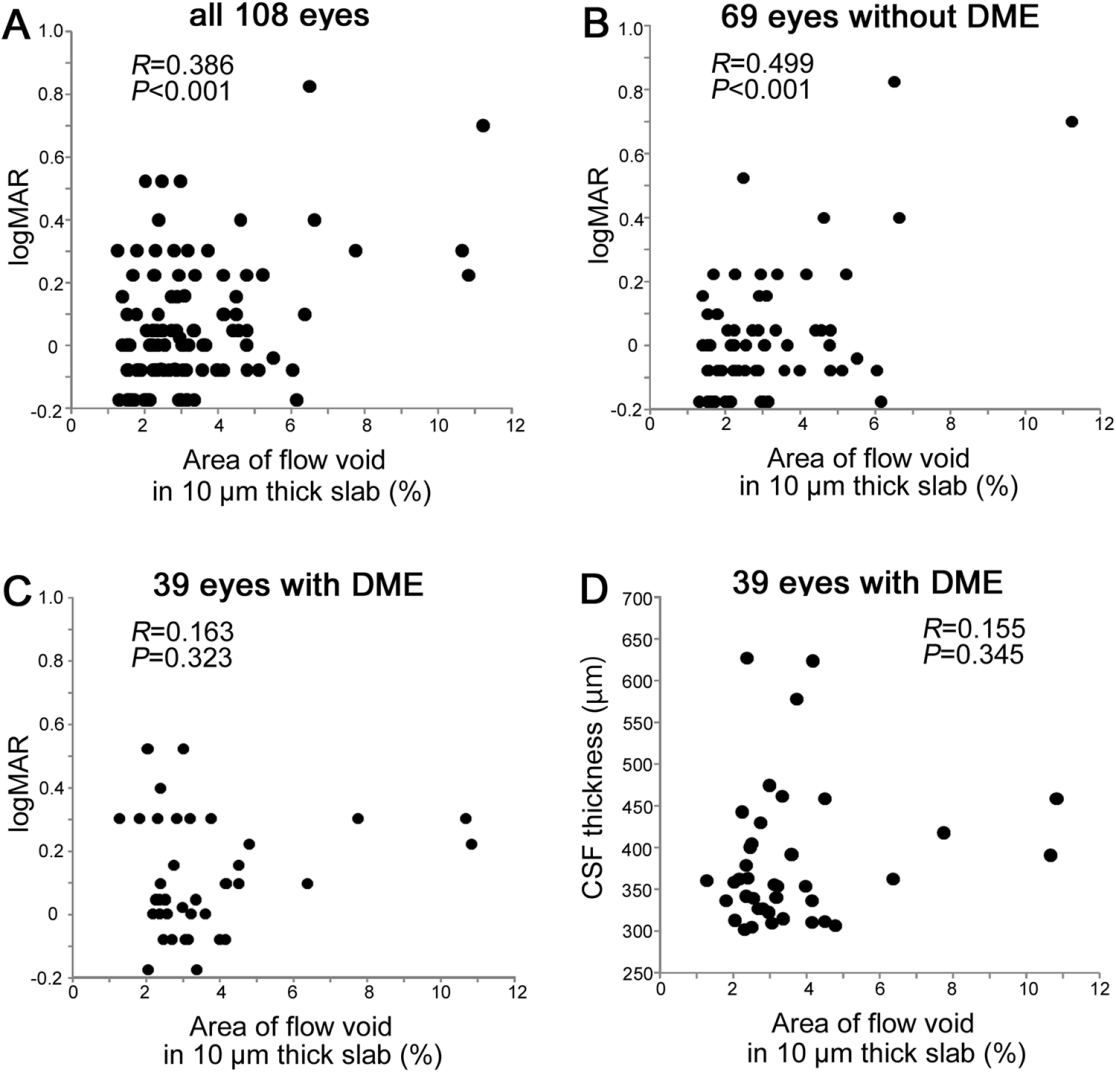
Association between logMAR VA and the areas of flow void in the choriocapillaris layer. There is a modest correlation between logMAR VA and the non-flow areas within the CSF in all 108 eyes (**A**) and in the 69 eyes without center-involved DME (**B**). (**C**,**D**) The areas of flow void in the CSF are not associated with either logMAR VA or CSF thickness in the 39 eyes with center-involved DME.

Further statistical analyses showed that the logarithm of the minimum angle of resolution visual acuity (logMAR VA) was modestly associated with the non-flow areas within the CSF in either all 108 eyes or 69 eyes without center-involved DME (Fig. 7A,B). By contrast, no relationship was observed between the areas of flow void and logMAR VA in eyes with center-involved DME (Fig. 7C). No differences in the non-flow areas were noted between eyes with and without center-involved DME (*p* = 0.150), and the CSF thickness was not correlated with the non-flow areas in eyes with center-involved DME (Fig. 7D).

We compared the areas of flow void in the regions with and without intact ellipsoid zone of the photoreceptor (EZ) lines in 12 eyes with ischemic maculopathy among 108 eyes. Within the non-flow areas in the deep retinal capillaries, the regions without intact EZ lines had greater non-flow areas in the choriocapillaris layer than those with intact EZ lines (6.28 ± 3.34% vs. 2.00 ± 1.10%; *p* < 0.001) (Fig. 5).

## Discussion

This retrospective study showed larger areas of flow void in the inner choroid in eyes with moderate NPDR, severe NPDR, and PDR than those with no apparent retinopathy. Since the choroid nourishes the outer retinal layers, the reduced decorrelation signals might represent the disrupted blood flow in the choroid, which would contribute to retinal ischemia and concomitant expression of VEGF^10, 33^. Intriguingly, the non-flow areas in the choriocapillaris layer were associated with poorer VA and disrupted photoreceptor cells, thus indicating their clinical relevance and suggesting the pathological contribution of disrupted choroidal circulation to neuroglial impairment in DR.

Histological publications showed that the intercapillary spaces in the choriocapillaris layer in the macula were much smaller than those in the retina^7, 34^. Since the lateral resolution on en-face OCTA images was not high, we could not definitively recognize the choroidal capillaries but could recognize the retinal capillaries on 3 × 3-mm en-face OCTA images^8^. This encouraged us to investigate the areas of flow void as an index of diabetes-induced dropout of capillaries in the choriocapillaris layer rather than the vascular structure per se^11, 13^. A choriocapillaris lobule in the macula with a diameter that ranges from dozens to hundreds of micrometers is fed by an arteriole, and the dark spots of a similar size on OCTA images might represent disrupted blood flow in this unit^9^. The non-flow areas with extended and undefined shapes might correspond to the histologic capillary dropout in the choriocapillaris layer.

OCTA showed that among several diseases with flow void in the inner choroid, geographic atrophy (GA) had significantly attenuated decorrelation signals in the choriocapillaris layer^32^. Eyes with DR and GA appeared to share these characteristics in vascular morphology. By contrast, the boundary was indefinite, and the decorrelation signals were mildly attenuated in the most choroidal lesions in DR compared with GA^35, 36^. Theoretically, OCTA does not allow us to know the definite absence of *in vivo* blood flow, and the decorrelation signals represent the limited range of flow velocity^36^. Further advances in fundus imaging technology should elucidate the structural and functional changes in choroidal vessels more definitively.

The areas of flow void were extended in the choriocapillaris layers as DR progressed. Systemic factors, including hyperglycemia and hypertension, might be common upstream regulators of both retinal and choroidal vascular lesions^37, 38^. Another possibility might be that VEGF from the retinas in DR increases Intercellular Adhesion Molecule (ICAM) expression, and concomitant leukostasis exacerbates capillary nonperfusion in the choriocapillaris layer^39, 40^. Alternatively, the circulatory disturbance in the choroid might contribute to DR progression. The outer retinal layers are nourished by the choroidal circulation in healthy eyes. Disrupted blood flow in the choroid would contribute to outer retinal ischemia, which would promote VEGF expression and subsequent DR progression^33^. VEGF expression is increased mainly in ganglion cells, astrocytes, Müller cells, and RPE in diabetic retinas, and a future study should evaluate whether VEGF is up-regulated in the photoreceptors in eyes with disrupted choroidal circulation^41^. Another explanation might be that diabetes-induced damage to the RPE, which contributes to the maintenance of capillaries in the choriocapillaris layer, might promote capillary dropout in the inner choroid^42–44^.

A recent publication reported the association between deep retinal capillary nonperfusion and photoreceptor disruption in diabetic macular ischemia^45^. We found that disrupted choroidal circulation is associated with photoreceptor damage and visual impairment within the areas of the deep retinal capillary nonperfusion. A deficiency in oxygen or nutrients from the chorioretinal capillaries could contribute to photoreceptor damage. Three of 12 eyes had center-involved DME, which could also induce photoreceptor damage and be a confounding factor for the analyses in this study. Further study of a large cohort should be planned to elucidate the processes involved in the damage to photoreceptors and choroidal circulation.

Classical fundus imaging using fluorescent dyes delineated hypofluorescence in the choroidal flush phase of fluorescein angiography or the early phase of indocyanine green angiography in DR, suggesting disruption in choroidal blood flow^46, 47^. These modalities provide two-dimensional data, whereas three-dimensional OCTA images specifically depict the motion contrast images in the inner choroidal layer. Another modality, laser Doppler flowgraphy, allows the measurement of flow velocity, although OCTA images show a limited range of velocity^33, 36^. Comparative studies would improve the understanding of disrupted choroidal circulation in DR.

The current study had several limitations, e.g., its retrospective nature and the small number of cases. We carefully excluded the areas with projection artifacts from the retinal vessels and shadow cast by retinal lesions with higher OCT reflectivity; however, it is unclear how these artifacts might influence the non-flow areas in the inner choroidal layers^48^. We had to recognize the limited generalizability because we used one of the major OCTA devices to image structures in Asian patients who contain higher amounts of pigment in the RPE and choroid. Another population-based study should be planned to compare the flow void between nondiabetic and diabetic subjects and to evaluate the reproducibility in other races, including other participants in whom systemic parameters are normalized, and using other devices.

In conclusion, we documented the lesions with attenuated decorrelation signals in the inner choroid in DR and their association with visual impairment and photoreceptor damage, suggesting the clinical relevance and pathophysiology of choroidal circulation in eyes with DR.

## Methods

### Patients

We retrospectively reviewed 108 consecutive eyes of 66 patients with DR who visited the Department of Ophthalmology of Kyoto University Hospital from February 2015 to August 2016. Eyes with DR were included when OCTA images of sufficient quality were acquired using Optovue RTVue XR Avanti (Optovue, Fremont, CA). The exclusion criteria were the presence of other chorioretinal diseases, glaucoma or ocular hypertension; an axial length shorter than 22.0 mm or longer than 26.0 mm; intraretinal or preretinal hemorrhages or hard exudates with diameters larger than one-third of the disc diameter within the central 3 × 3-mm square on the color fundus photograph; a history of any intervention for macular lesions; intraocular surgery other than cataract extraction; and cataract surgery within 6 months of study enrollment. We excluded eyes with a history of photocoagulation of the macular area because the decorrelation signals in the choriocapillaris layer were attenuated after this treatment^49^. To guarantee image quality, we also excluded eyes with a signal strength index (SSI) score of 60 or less^50^. Thirty-nine eyes had center-involved DME, which was determined by two-dimensional mapping constructed using the same dataset. According to the previously reported equation, the cutoff value for the CSF thickness was 299.52 µm on Optovue images, which corresponds to 250 µm on OCT images obtained by Stratus OCT (Carl Zeiss Meditec AG, Oberkochen, Germany)^51, 52^. We thus determined that the eyes with a CSF thickness exceeding 299.52 µm had center-involved DME in the current study.

All research and measurements adhered to the tenets of the Declaration of Helsinki. The Kyoto University Graduate School and Faculty of Medicine Ethics Committee approved the study protocol. All participants provided written informed consent before inclusion in the study.

### OCTA

We performed a comprehensive ophthalmic examination including the color fundus photography (TRC-NW8F; Topcon Corp, Tokyo, Japan) and the measurement of the axial length using partial coherence interferometry (IOLMaster, Carl Zeiss Meditec AG). The best-corrected decimal VA was converted to logMAR VA. OCTA images in the 3 × 3-mm square centered on the fovea were obtained using the Optovue RTVue XR Avanti (Fig. 1). Given the high A-scan speed of 70,000 scans/second and a light source of approximately 840 nm, this instrument delineates motion-dependent decorrelation signals using the split-spectrum amplitude decorrelation angiography algorithm. Briefly, three-dimensional scans are comprised of two consecutive B-scans (M-B frames) at a fixed position. The calculation of the decorrelation between the sequential images enables the detection of the motion of the blood cells and concomitant construction of a motion contrast AngioFlow image.

We applied segmentation processes to create the en-face OCTA images in the choroidal capillary slab, the retinal superficial and deep capillary plexus layers, and the avascular outer retina and the en-face structural OCT images in the RPE slab using the manufacturer’s software. The choroidal capillary slab images using the default setting in the manufacturer’s software corresponded to the inner choroidal slab images extending from 31 to 59 μm posterior to the RPE-Bruch membrane complex segmentation. According to a publication documenting *in vivo* thicknesses in the choriocapillaris-Sattler’s layer complex and Haller’s layer, the choroidal capillary slab might correspond to parts of the choriocapillaris-Sattler’s layer complex in most eyes^53^. Additionally, we prepared the ~10-μm-thick en-face images in the choriocapillaris layer (from 31 to 40 μm posterior to the RPE-Bruch membrane complex) according to a recent publication^54^. The corresponding structural OCT images were also created to assess shadow artifacts. The retinal superficial or deep slab OCTA images showed the superficial layer from the inner boundary 3 μm beneath the internal limiting membrane (ILM) to the outer boundary 15 μm beneath the inner plexiform layer (IPL) or the deep layer from the inner boundary 15 μm beneath the IPL to the outer boundary 70 μm beneath the IPL. The en-face images in the avascular outer retina were from the inner boundary 70 μm below the IPL to the outer boundary located 30 μm below the RPE reference. Given that most shadow artifacts from the intraretinal lesions are clearly depicted in the RPE, we created an en-face structural OCT slab (~28 μm thick) at the level of the RPE as described recently^50^. These images were exported into ImageJ (NIH, Bethesda, MD) for image processing and analyses.

We then quantified the areas of flow void in the choroidal capillary slab in individual subfield of the modified ETDRS grid, including the central subfield (1 mm diameter) and the parafoveal quadrants (superior, inferior, nasal, and temporal subfields from 1 mm to 2.5 mm diameter), after the removal of the areas with projection artifacts and shadow artifacts as described recently^50^. Histologic publications suggested that this instrument cannot detect the specific morphology of the normal vascular network in the choriocapillaris layer within the macular area^7–9^. We thus evaluated the intensity of the decorrelation signals as the index of blood flow but not the vascular structure per se in the choriocapillaris layer. The preliminary study showed that the decorrelation signals are occasionally increased in the dilated capillaries around the nonperfused areas, which might incorrectly increase the average signal intensity. We therefore determined the mean decorrelation signals in the avascular outer retina as the cutoff value and measured the areas with decorrelation signals below the threshold to assess the disrupted blood flow.

We first calculated the mean levels of decorrelation signals in the avascular outer retina of every eye individually and used them as the individual threshold of the flow void in two en-face OCTA images of the choroidal capillary layer, i.e., the inner choroidal slab image using the default setting of the manufacturer’s software (from 31 to 59 μm posterior to the RPE-Bruch membrane complex segmentation) and the 10-μm-thick choriocapillaris slab image (from 31 to 40 μm posterior to the RPE-Bruch membrane complex) (Fig. 1I)^50^. We further created the binary images of the en-face OCTA image in the retinal superficial layer and the structural OCT image in the RPE using the global threshold as described recently (Fig. 1G,H)^50^. We then removed the projection artifacts from the retinal superficial vessels and the shadow artifacts from the preretinal or intraretinal hyperreflective lesions, e.g., microaneurysms, hemorrhages and hard exudates (Fig. 1K), before we measured the areas of flow void and calculated the percentage of the non-flow areas in the total areas without artifacts.

We further investigated the relationship between photoreceptor damage and the non-flow areas in the choriocapillaris layer in eyes with ischemic maculopathy. We first determined ischemic maculopathy of grade 3 or 4 according to the modified methods described by the ETDRS Report Number 11^55^. We merged the en-face OCTA images in the retinal superficial and deep capillary layers (Fig. 5D) using the colour merge function in the analysis plugin WCIF-ImageJ (http://www.uhnresearch.ca/facilities/wcif/imagej/). Ischemic maculopathy was defined on the merged OCTA images instead of FA images based on the agreement of two independent masked graders. We selected and further evaluated 12 eyes with the outline of the FAZ destroyed for one-half or more of the original circumference^55^. A recent publication demonstrated the association between photoreceptor disruption and deep retinal capillary nonperfusion in diabetic eyes^45^, prompting us to investigate the relationship between the EZ status and the flow void in the choriocapillaris layer within the nonperfused areas in the deep retinal capillary layer. We prepared the binary images of the non-flow areas in the choriocapillaris layer and artifacts (Fig. 5E,F). The regions without intact EZ lines were determined on the B-scan images along 10 transverse lines (17th, 47th, 77th, 107th, 137th, 167th, 197th, 227th, 257th, and 287th lines from the most superior line; Fig. 5G,I,K,L)^14^. The transverse length of the areas of flow void and those without artifacts were measured within the corresponding regions (Fig. 5K,L). We then summed the transverse lengths along all 10 lines and calculated the percentages of the areas of flow void. Similarly, we evaluated the non-flow areas in the regions with intact EZ lines (Fig. 5M,N).

### Statistical analysis

The data are expressed as the mean ± SD. After the Kolmogorov–Smirnov test was applied to confirm that the dataset was normally distributed, the significant differences were evaluated using Student’s t-test or analysis of variance with Bonferroni correction, and the correlation was assessed using Pearson’s correlation coefficient.

## References

[1] Yau JW (2012). Global prevalence and major risk factors of diabetic retinopathy. Diabetes Care.

[2] Early Treatment Diabetic Retinopathy Study research group (1985). Photocoagulation for diabetic macular edema. Early Treatment Diabetic Retinopathy Study report number 1. Early Treatment Diabetic Retinopathy Study research group. Arch. Ophthalmol..

[3] Diabetic Retinopathy Vitrectomy Study (1985). Two-year course of visual acuity in severe proliferative diabetic retinopathy with conventional management. Diabetic Retinopathy Vitrectomy Study (DRVS) report #1. Ophthalmology.

[4] Cunningham ET (2005). A phase II randomized double-masked trial of pegaptanib, an anti-vascular endothelial growth factor aptamer, for diabetic macular edema. Ophthalmology.

[5] Gardner TW, Antonetti DA, Barber AJ, LaNoue KF, Levison SW (2002). Diabetic retinopathy: more than meets the eye. Surv. Ophthalmol..

[6] Antonetti DA, Klein R, Gardner TW (2012). Diabetic retinopathy. N. Engl. J. Med..

[7] Olver JM (1990). Functional anatomy of the choroidal circulation: methyl methacrylate casting of human choroid. Eye (Lond).

[8] Choi W (2013). Choriocapillaris and choroidal microvasculature imaging with ultrahigh speed OCT angiography. PLoS One.

[9] Zouache MA, Eames I, Klettner CA, Luthert PJ (2016). Form, shape and function: segmented blood flow in the choriocapillaris. Sci. Rep..

[10] Bhutto I, Lutty G (2012). Understanding age-related macular degeneration (AMD): relationships between the photoreceptor/retinal pigment epithelium/Bruch’s membrane/choriocapillaris complex. Mol. Aspects Med..

[11] Hidayat AA, Fine BS (1985). Diabetic choroidopathy. Light and electron microscopic observations of seven cases. Ophthalmology.

[12] Fryczkowski AW, Hodes BL, Walker J (1989). Diabetic choroidal and iris vasculature scanning electron microscopy findings. Int. Ophthalmol..

[13] Cao J, McLeod S, Merges CA, Lutty GA (1998). Choriocapillaris degeneration and related pathologic changes in human diabetic eyes. Arch. Ophthalmol..

[14] Murakami T (2012). Optical coherence tomographic reflectivity of photoreceptors beneath cystoid spaces in diabetic macular edema. Invest. Ophthalmol. Vis. Sci..

[15] Spaide RF, Koizumi H, Pozzoni MC (2008). Enhanced depth imaging spectral-domain optical coherence tomography. Am. J. Ophthalmol..

[16] Esmaeelpour M (2011). Mapping choroidal and retinal thickness variation in type 2 diabetes using three-dimensional 1060-nm optical coherence tomography. Invest. Ophthalmol. Vis. Sci..

[17] Adhi M, Brewer E, Waheed NK, Duker JS (2013). Analysis of morphological features and vascular layers of choroid in diabetic retinopathy using spectral-domain optical coherence tomography. JAMA Ophthalmol.

[18] Kim JT, Lee DH, Joe SG, Kim JG, Yoon YH (2013). Changes in choroidal thickness in relation to the severity of retinopathy and macular edema in type 2 diabetic patients. Invest. Ophthalmol. Vis. Sci..

[19] Gerendas BS (2014). Three-dimensional automated choroidal volume assessment on standard spectral-domain optical coherence tomography and correlation with the level of diabetic macular edema. Am. J. Ophthalmol..

[20] Querques G (2012). Enhanced depth imaging optical coherence tomography in type 2 diabetes. Invest. Ophthalmol. Vis. Sci..

[21] Yiu G, Manjunath V, Chiu SJ, Farsiu S, Mahmoud TH (2014). Effect of anti-vascular endothelial growth factor therapy on choroidal thickness in diabetic macular edema. Am. J. Ophthalmol..

[22] Rayess, N. *et al*. Baseline choroidal thickness as a predictor for response to anti-vascular endothelial growth factor therapy in diabetic macular edema. *Am*. *J*. *Ophthalmol*. **159**, 85–91 e81–83 (2015). 10.1016/j.ajo.2014.09.033 25261844

[23] Sonoda S (2014). Effect of intravitreal triamcinolone acetonide or bevacizumab on choroidal thickness in eyes with diabetic macular edema. Invest. Ophthalmol. Vis. Sci..

[24] Xu J (2013). Subfoveal choroidal thickness in diabetes and diabetic retinopathy. Ophthalmology.

[25] Ferrara D, Waheed NK, Duker JS (2016). Investigating the choriocapillaris and choroidal vasculature with new optical coherence tomography technologies. Prog. Retin. Eye Res..

[26] Murakami T (2016). *In Vivo* Choroidal Vascular Lesions in Diabetes on Swept-Source Optical Coherence Tomography. PLoS One.

[27] Mariampillai A (2008). Speckle variance detection of microvasculature using swept-source optical coherence tomography. Opt. Lett..

[28] Jia Y (2012). Split-spectrum amplitude-decorrelation angiography with optical coherence tomography. Opt. Express.

[29] Schwartz DM (2014). Phase-variance optical coherence tomography: a technique for noninvasive angiography. Ophthalmology.

[30] Spaide RF, Klancnik JM, Cooney MJ (2015). Retinal vascular layers imaged by fluorescein angiography and optical coherence tomography angiography. JAMA Ophthalmol.

[31] Spaide RF (2016). Choriocapillaris Flow Features Follow a Power Law Distribution: Implications for Characterization and Mechanisms of Disease Progression. Am. J. Ophthalmol..

[32] Pellegrini M (2016). Dark Atrophy: An Optical Coherence Tomography Angiography Study. Ophthalmology.

[33] Nagaoka T (2004). Alteration of choroidal circulation in the foveal region in patients with type 2 diabetes. Br. J. Ophthalmol..

[34] Chan G (2012). Quantitative morphometry of perifoveal capillary networks in the human retina. Invest. Ophthalmol. Vis. Sci..

[35] Jia Y (2015). Quantitative optical coherence tomography angiography of vascular abnormalities in the living human eye. Proc. Natl. Acad. Sci. USA.

[36] Choi W (2015). Ultrahigh-Speed, Swept-Source Optical Coherence Tomography Angiography in Nonexudative Age-Related Macular Degeneration with Geographic Atrophy. Ophthalmology.

[37] The effect of intensive treatment of diabetes on the development and progression of long-term complications in insulin-dependent diabetes mellitus. The Diabetes Control and Complications Trial Research Group. *N*. *Engl*. *J*. *Med*. **329**, 977–986 (1993). 10.1056/NEJM199309303291401 8366922

[38] Group AS (2010). Effects of medical therapies on retinopathy progression in type 2 diabetes. N. Engl. J. Med..

[39] Miyamoto K (2000). Vascular endothelial growth factor (VEGF)-induced retinal vascular permeability is mediated by intercellular adhesion molecule-1 (ICAM-1). Am. J. Pathol..

[40] Campochiaro PA, Wykoff CC, Shapiro H, Rubio RG, Ehrlich JS (2014). Neutralization of vascular endothelial growth factor slows progression of retinal nonperfusion in patients with diabetic macular edema. Ophthalmology.

[41] Murata T (1996). The relation between expression of vascular endothelial growth factor and breakdown of the blood-retinal barrier in diabetic rat retinas. Lab. Invest..

[42] Blaauwgeers HG (1999). Polarized vascular endothelial growth factor secretion by human retinal pigment epithelium and localization of vascular endothelial growth factor receptors on the inner choriocapillaris. Evidence for a trophic paracrine relation. Am. J. Pathol..

[43] Kurihara T, Westenskow PD, Bravo S, Aguilar E, Friedlander M (2012). Targeted deletion of Vegfa in adult mice induces vision loss. J. Clin. Invest..

[44] Simo R, Villarroel M, Corraliza L, Hernandez C, Garcia-Ramirez M (2010). The retinal pigment epithelium: something more than a constituent of the blood-retinal barrier–implications for the pathogenesis of diabetic retinopathy. J. Biomed. Biotechnol..

[45] Scarinci F, Nesper PL, Fawzi AA (2016). Deep Retinal Capillary Nonperfusion Is Associated With Photoreceptor Disruption in Diabetic Macular Ischemia. Am. J. Ophthalmol..

[46] Freyler H, Prskavec F, Stelzer N (1986). [Diabetic choroidopathy–a retrospective fluorescein angiography study. Preliminary report]. Klin. Monbl. Augenheilkd..

[47] Shiragami C, Shiraga F, Matsuo T, Tsuchida Y, Ohtsuki H (2002). Risk factors for diabetic choroidopathy in patients with diabetic retinopathy. Graefes Arch. Clin. Exp. Ophthalmol..

[48] Spaide RF, Fujimoto JG, Waheed NK (2015). Image Artifacts in Optical Coherence Tomography Angiography. Retina.

[49] Cole ED (2016). Visualization of Changes in the Choriocapillaris, Choroidal Vessels, and Retinal Morphology After Focal Laser Photocoagulation Using OCT Angiography. Invest. Ophthalmol. Vis. Sci..

[50] Nesper PL, Soetikno BT, Fawzi AA (2017). Choriocapillaris Nonperfusion is Associated With Poor Visual Acuity in Eyes With Reticular Pseudodrusen. Am. J. Ophthalmol..

[51] Bressler SB (2015). Reproducibility of Optovue RTVue Optical Coherence Tomography Retinal Thickness Measurements and Conversion to Equivalent Zeiss Stratus Metrics in Diabetic Macular Edema. Transl. Vis. Sci. Technol..

[52] Browning DJ (2007). Relationship between optical coherence tomography-measured central retinal thickness and visual acuity in diabetic macular edema. Ophthalmology.

[53] Branchini LA (2013). Analysis of choroidal morphologic features and vasculature in healthy eyes using spectral-domain optical coherence tomography. Ophthalmology.

[54] Alten F, Heiduschka P, Clemens CR, Eter N (2016). Exploring choriocapillaris under reticular pseudodrusen using OCT-Angiography. Graefes Arch. Clin. Exp. Ophthalmol..

[55] Early Treatment Diabetic Retinopathy Study Research Group. Classification of diabetic retinopathy from fluorescein angiograms. ETDRS report number 11. Early Treatment Diabetic Retinopathy Study Research Group. *Ophthalmology* **98**, 807–822 (1991). 2062514

